# *WNT5A* is a putative epi‐driver of prostate cancer metastasis to the bone

**DOI:** 10.1002/cam4.70122

**Published:** 2024-08-20

**Authors:** Emma J. Wilkinson, Kelsie Raspin, Roslyn C. Malley, Shaun Donovan, Louise M. Nott, Adele F. Holloway, Joanne L. Dickinson

**Affiliations:** ^1^ Tasmanian School of Medicine University of Tasmania Hobart Tasmania Australia; ^2^ Menzies Institute for Medical Research University of Tasmania Hobart Tasmania Australia; ^3^ Anatomical Pathology Royal Hobart Hospital Hobart Tasmania Australia; ^4^ Diagnostic Services Sonic Healthcare Hobart Tasmania Australia; ^5^ Icon Cancer Centre Hobart Tasmania Australia; ^6^ Oncology and Haematology Royal Hobart Hospital Hobart Tasmania Australia

**Keywords:** DNA methylation, epigenetic driver, gene expression, metastasis, prostate cancer

## Abstract

**Background:**

Current diagnostic tools are unable to distinguish low‐grade indolent prostate cancer (PrCa) from that with a propensity to become metastatic and/or lethal. Recent evidence suggests that reprogramming of the transcriptome may drive the metastatic phenotype, and that this reprogramming is controlled, at least in part, by epigenetic changes to the DNA of cancer cells, including methylation. These changes, referred to as ‘epigenetic drivers,’ have previously been associated with cancer cell survival.

**Methods:**

Here, using Illumina Methylation EPIC array data of paired primary PrCa and metastatic bone samples, we identified *WNT5A* as a putative epi‐driver of PrCa metastasis to the bone, which was further validated in vitro.

**Results:**

Significantly higher *WNT5A* methylation was observed in primary PrCa samples and 22Rv1 cells compared to metastatic bone samples and PC‐3 cells. This higher methylation was associated with significantly lower *WNT5A* gene expression.

**Conclusion:**

Given the limited effective therapies available for metastatic cancer sufferers, particularly those whose disease has metastasised to the bone, *WNT5A* presents as a potential putative target for therapy.

## Graphical abstract

We performed epigenomic analysis in a rare dataset of paired primary prostate and metastatic bone tumours. Our study identified methylation changes in *WNT5A* between primary and metastatic tumours, and these were shared among individuals. Identified epigenetic alterations were then followed up in in vitro cell line models of prostate cancer and publicly available databases.

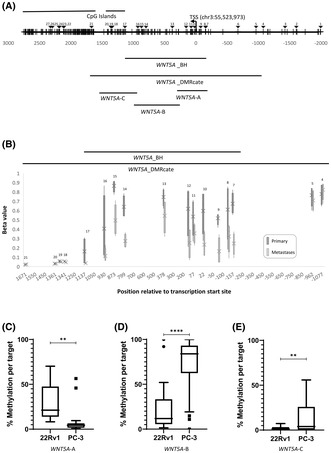

## 1 INTRODUCTION

In prostate cancer (PrCa), like many solid malignancies, mortality is largely due to progression of the primary tumour to metastatic disease. Localised PrCa has a high 5‐year survival rate of over 90%,^1^ however, once metastasis occurs this dramatically decreases to 24.5%.^2^ PrCa preferentially metastasises to the bone,^3^ which is associated with an even lower 5‐year survival rate of just 3%.^4^ Whilst multiple molecular pathways have been implicated in metastasis, the key mechanisms driving preferential spread of PrCa cells to survive and proliferate in the bone, remain elusive. Given that bone metastasis is a strong indicator of disease prognosis and those living with metastatic cancer suffer significant disability and pain, a better understanding of the pathways through which PrCa metastasis occurs is urgently needed.

Despite an increase in PrCa diagnoses and an improvement in the 5‐year survival rate following diagnosis, there has been no consistent decrease in PrCa mortality associated with increased diagnosis,^5^ and this is likely due to a sustained number of men progressing to metastatic disease. There is no cure once the tumour has metastasised to the bone, and current therapies are both limited and largely ineffective. As such, they are mainly palliative, focusing on delaying bone destruction to preserve physical function and manage pain.^6^

The majority of research has focussed on the genetic and epigenetic changes associated with the initiation and development of primary prostate tumours, which are frequently diagnosed as low‐grade, and often slow growing tumours (indolent). However, high‐grade tumours that have the potential to become aggressive, and even metastatic, are those of significant clinical concern. Particularly those that spread to the bone and become resistant to treatment, inevitably leading to death. There has been intensive work performed in attempts to identify those tumours with a metastatic capability by curating the genetic characteristics of aggressive primary tumours and cancer stem cells populations.^7^ Although a myriad of molecules have been found to participate in metastatic processes; which of these factors drive metastatic‐specific capabilities remains unclear.

It has been suggested that primary tumour cells undergo transcriptional re‐programming giving rise to ‘metastasis capable’ cells,^8^ which suggests that altered gene regulation mediated, at least in part, by epigenetic mechanisms, such as DNA methylation, may contribute to the bone ‘metastasis capable’ phenotype.^9^ For example, in paired primary and metastatic samples from pancreatic ductal adenocarcinoma patients, epigenomic signatures of locally spreading lymph node lesions are molecularly distinct from distant metastases.^10^ In PrCa, a small number of studies using relatively few paired primary and metastatic tumours reveal distinctive methylation signatures between the primary and distal sites.^11^ Notably, such epigenetic signatures may have clinical applications in predicting disease aggressiveness in patient samples.

A refocus of our research effort on the genes and pathways facilitating PrCa progression to metastatic disease will allow for the identification of putative drug targets and an eventual benefit to disease prognosis. It is also hoped that characterisation of these molecular drivers may allow for better identification of tumours that are likely to become metastatic (at primary diagnosis), leading to a decrease in unnecessary treatment of indolent tumours. Here, we utilised a rare cohort of paired primary and metastatic bone samples to identify potential epigenetic drivers (epi‐drivers) of secondary PrCa growth in bone. As a result, we highlighted *WNT5A* as a key putative epi‐driver of PrCa bone metastasis.

## 2 MATERIALS AND METHODS

### 2.1 Cell culture

The PC‐3 (RRID:CVCL_0035) and 22Rv1 (RRID:CVCL_1045) cell lines were obtained from the European Collection of Authenticated Cell Cultures (ECACC, UK), and determined to be mycoplasma free. Both cell lines were maintained in Roswell Park Memorial Institute 1640 medium (RPMI 1640; Sigma‐Aldrich Corporation) containing 10% foetal calf serum and penicillin/streptomycin solution in a humidified incubator at 37°C and 5% CO_2_. Both cell lines were authenticated using short tandem repeat profiling at the Australian Genome Research Facility (AGRF) within the last 3 years.

For experiments in which cells were treated with *5‐aza‐2′‐deoxycytidine* (AzaC), cells were grown to 50% confluent (PC‐3) and 20%–25% confluent (22Rv1), respectively and treated with 0.3 μM final concentration of AzaC or DMSO (vehicle control) for one population doubling time (24 h for PC‐3, 48 h for 22Rv1) in RPMI media. After one population doubling time, AzaC was removed and cells were grown for another two population doubling times in fresh RPMI before being harvested.

### 2.2 Isolation of DNA from prostate tumour samples and cell lines

Archived pathology tumour blocks were obtained from pathology laboratories in Tasmania using a waiver of consent, under ethics approval from the University of Tasmania's Human Research Ethics Committee (H0020219). Formalin fixed paraffin embedded (FFPE) tissue samples (5 μm sections) were dewaxed, incubated at 56°C in Proteinase K for 24 h and DNA extracted using the QIAamp DNA FFPE tissue Kit (QIAGEN), according to the manufacturer's instructions.

DNA was extracted from frozen cell pellets using the Accuprep® Genomic DNA Extraction Kit (Bioneer), following the manufacturer's protocol. Alternatively, DNA was extracted from frozen cell pellets using the QIAamp DNA Blood Mini Kit (QIAGEN), according to the manufacturer's protocol. Genomic DNA (500 ng) was bisulphite converted using the EZ DNA Methylation‐Gold Kit, according to the manufacturer's instructions (Zymo research, USA).

### 2.3 Methylation EPIC array analysis

DNA methylation in the FFPE samples was quantified using the Illumina Infinium MethylationEPIC v2.0 BeadChip array (EPIC array) at the AGRF and analysed using Illumina's GenomeStudio v2011.1 with Methylation module 1.9.0 software and the Illumina MethylationEPIC_v‐1‐0_B3 manifest file, as we have previously described.^12^

### 2.4 Bioinformatic analysis of paired patient samples

Initial quality control and analysis was conducted as we have previously described,^12^ with the following alterations: only probes with a detection *p*‐value of <0.01 and probes with a bead count of ≥3 in all samples were included for analysis. Both *β*‐values and *M*‐values were extracted with *M*‐values being used for further analysis. The resulting dataset consisted of 772,122 CpG sites.

Subsequently, two methods were utilised to identify differentially methylated regions (DMRs) between primary prostate and metastatic lesions, *bumphunter* ^13^ and *DMRcate.* ^14^ The initial 772,122 CpG sites previously mentioned, were used for *bumphunter* analysis. Additionally, for analysis with *DMRcate*, cross‐reactive probes were removed from the analysis. The resulting dataset consisted of 751,048 CpG sites.

For both analyses, data were divided into ‘localised tumour’ and ‘bone metastasis’ groups. For analysis with *bumphunter*, *M*‐values were processed through the *bumphunter* function (version 1.20.0)^13^ of *minfi* to identify regions of differential methylation between the two groups, with the following parameters: the cut‐off for percent difference in methylation was set at 90% and permutations were set to 10. For analysis with DMRcate, *M*‐values were processed through the *DMRcate* function (version 1.20.0)^14^ of *minfi* to identify regions of differential methylation between the two allocated groups with the following parameters: the *p*‐value cutoff was set to 0.01, the parameter lambda to 1000 and the parameter C to 2. These analyses with *bumphunter* and *DMRcate* yielded 8221 and 8934 significantly DMRs (*p* < 0.05), respectively. The genomic region covered by the DMRs from each analysis were annotated with *annotatr* (version 1.10.0)^15^ to identify which gene/s they were associated with, and their relation to CpG islands. Of the 8221 DMRs identified with *bumphunter* analysis, 5299 were associated with 3850 individual genes. Of the 8934 DMRs identified with *DMRcate* analysis, 5873 regions were associated with 5933 individual genes. The annotated lists of DMRs from both analyses were then cross‐referenced with one another using the ‘intersect’ function of *dplyr* (version 0.8.5).^16^

### 2.5 Targeted next generation bisulphite sequencing

Bisulphite converted cell line DNA (25 ng) was used to amplify three regions of interest with MyTaq HS™ (Bioline) using a Verti 96 well thermal cycler (Applied Biosystems, USA) under the following conditions: 95°C for 3 min followed by 45 cycles of 95°C for 30 s, 54°C for 30 s and 72°C for 30 s, followed by 72°C for 10 min, according to the manufacturer's instructions with the following alteration: 2% DMSO was added to the reaction mix. Primer sequences available upon request.

Libraries were prepared (1 ng PCR product) using the Nextera XT DNA Library Prep Kit (Illumina), according to the manufacturer's protocol with the following modifications: reactions were performed in one third of the recommended volume. Samples were barcoded using Set A and B of the Nextera XT Index v2 Kit (Illumina). Barcoded DNA fragments were pooled and sequenced on the MiSeq Illumina Platform using the MiSeq Reagent Nano v2 500 Cycle Kit (Illumina). FASTQ files were aligned to the hg19 reference genome, and percent methylation at each CpG site was determined using the web interface EPIC‐TABSAT v1.7120.^17^

### 2.6 RNA isolation and gene expression analysis

RNA (1 μg) was extracted from frozen cell pellets using the Direct‐Zol™ RNA MicroPrep Kit (Zymo Research), treated with DNase, and reverse transcribed using the iScript™ Reverse Transcription Supermix for RT‐qPCR Kit (Bio‐Rad) or the qScript cDNA Supermix Kit (Quantabio); all as per the manufacturer's directions. For quantitative PCR analysis, cDNA (50 ng) was amplified with the SensiFAST SYBR® No‐ROX Kit (Thermo Fisher Scientific), according to the manufacturer's instructions, using a 2‐step cycling protocol on a QuantStudio™ 3 Real‐Time PCR System (Applied Biosystems), under the following conditions: 2 min at 95°C, then 30 cycles of 95°C for 5 s then 60°C for 30 s, acquiring at the end of the 60°C step. Primer sequences are available upon request.

## 3 RESULTS

We hypothesised that analysis of methylation changes between primary and metastatic bone samples shared between individuals, may identify key epi‐drivers of PrCa progression from localised to metastatic disease. Four paired primary prostate tumour and metastatic bone samples were selected (clinicopathological information is listed in Table 1) and genome‐wide differences in DNA methylation were ascertained with the Illumina Methylation EPIC array.

**TABLE 1:** Clinicopathological characteristics of the paired primary PrCa and metastatic bone samples included in this study.

Sample ID	PSA	Gleason score	Primary tumour description	Metastasis description	Time between biopsy sampling (years)
A	10	7	Invasive	Poorly differentiated	5
B	17.2	9	Poorly differentiated, invasive	‐	3
C	‐	9	Poorly differentiated, invasive	‐	13
D	20	9	Poorly differentiated, invasive	‐	2

*Note*: Information not available on original pathology report. All FFPE sections were examined by a pathologist (RCM or SD).

Following quality control, the resulting dataset consisted of 772,122 CpG sites. The extracted *M*‐values were visualised on a multi‐dimensional scaling plot, which revealed that the major driver of variation is the individual, and not the sample type, i.e., primary prostate or bone lesion (Figure S1). This indicates that any DMRs identified that are shared between individuals are likely to be characteristic of metastatic disease progression more broadly.

To identify DNA methylation changes between paired primary tumour and metastatic bone samples, two methods were utilised to identify the DMRs, *bumphunter* ^13^ and *DMRcate.* ^14^ In total, the initial 772,122 CpG sites previously mentioned were used for *bumphunter* analysis, and following removal of cross‐reactive probes, 751,048 CpG sites were included in the *DMRcate* analysis. Cross referencing the resulting DMRs from both analyses revealed that 3436 (89%) of the genes identified in the *bumphunter* analysis were also identified in the *DMRcate* analysis. KEGG pathway analysis of these overlapping genes found that these genes were enriched in the following: metabolic pathways, pathways in cancer, PI3K‐Akt signalling pathway, MAPK signalling pathway, Human papillomavirus infection, and microRNAs in cancer, among other cancer‐related pathways. This analysis confirms that the identified DMRs are likely specific to cancer progression.

Finally, the top 100 DMR gene lists from both analyses were sorted in descending order of statistical significance. The top 10 significant DMRs from each analysis, shown in Tables S1 and S2, were then cross referenced to identify regions of interest. Cross‐referencing the top 10 DMR lists identified four common regions, of which three were associated with genes (*CD81*; chr11:2,397,201‐2,398,533, *ST6FALNAC1*; chr17:74,641,167‐74,641,294, *WNT5A*; chr3:55,522,301‐55,524,129), and one was in an intergenic region on chromosome 12, 12,200 bp upstream of *TBX3*. Given that *WNT5A* expression has been associated with multiple cancer types and many stages of disease,^18^ *WNT5A* was prioritised for follow‐up here.

### 3.1 *WNT5A* is differentially methylated in primary prostate tumours when compared to bone metastases from the same individual

**FIGURE 1:**
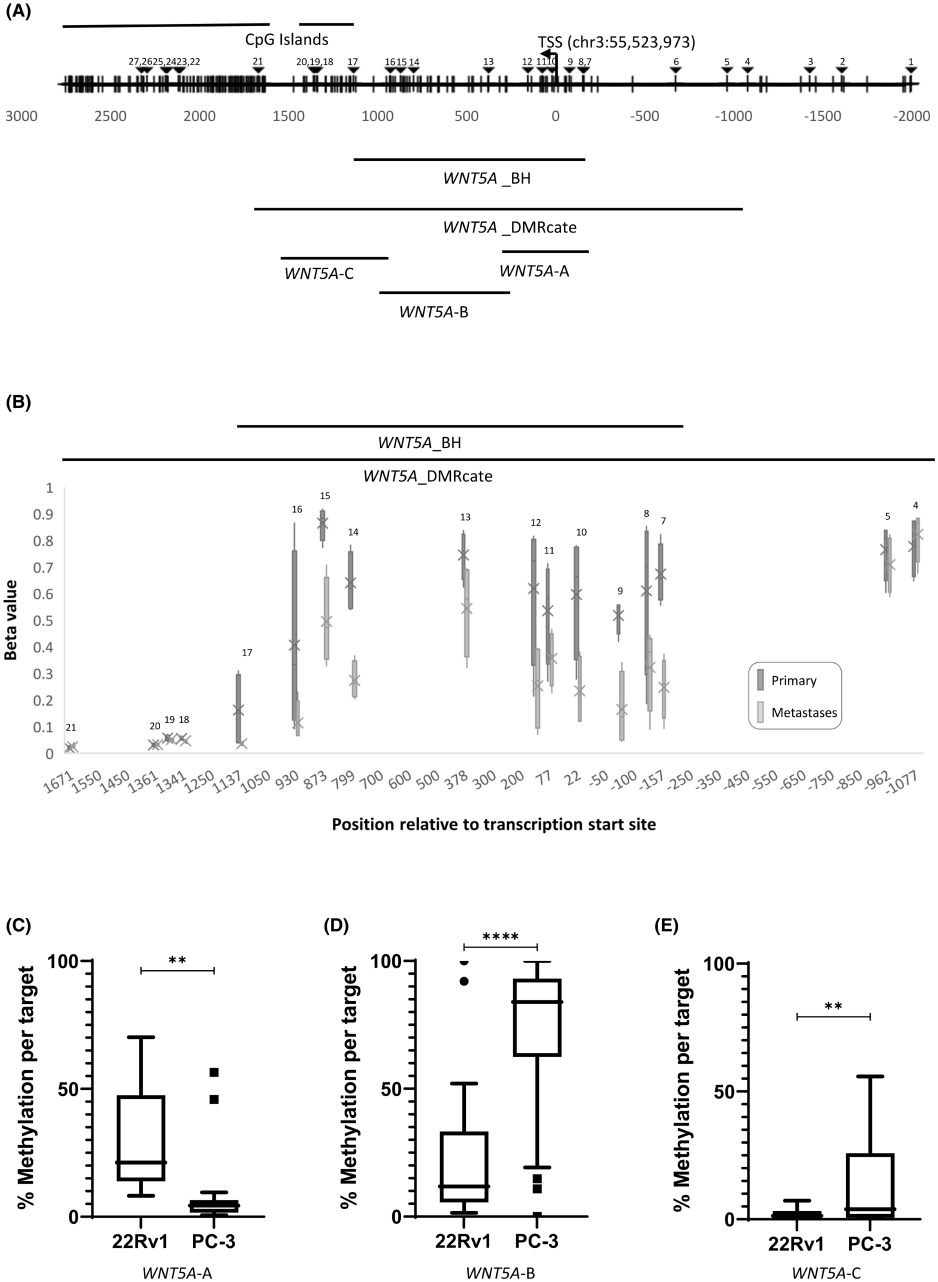
(A) Schematic representation of the promoter region of *WNT5A*. Transcription start site (TSS) at chr3: 55,523,973 (hg19) and CpG islands are indicated. CpG sites are indicated by vertical lines. CpG sites interrogated by the Illumina Methylation EPIC array are indicated by triangles and labelled 1–27. Region from *bumphunter* (WNT5A_BH) and *DMRcate* (WNT5A_DMRcate) analyses are indicated. Region amplified using primer sets WNT5A‐A, WNT5A‐B and WNT5A‐C and analysed by bisulphite sequencing are indicated. (B) *WNT5A* methylation in paired primary PrCa and metastatic bone samples. Methylation status of the 17 CpG sites within the significantly differentially methylated region found in *WNT5A* with DMRcate analysis in tumour (dark grey) and paired bone metastases (light grey) as determined using the EPIC array. The significantly differentially methylated region found by *bumphunter* analysis (11 CpGs) is indicated. CpG numbers correspond to (A). The box plots depict the spread of beta values for each CpG across the four patient samples in each group (primary vs. metastasis). The mean of the group is labelled with a cross. The beta values are taken from many reads of ‘methylated’ or ‘unmethylated’ to generate a percentage methylated (1 being fully methylated, 0 being unmethylated) per sample. CpG site #6 failed quality control. (C–E) Average *WNT5A* methylation across the region encompassed by primer sets WNT5A‐A (18 CpGs) (C), WNT5A‐B (30 CpGs) (D) and WNT5A‐C (20 CpGs) (E) in prostate cancer cells, 22Rv1 and PC‐3. Statistical significance was determined using a paired *t*‐test, **p < 0.01, ****p < 0.0001.

One of the most significant DMRs was situated across the transcription start site (TSS) of *WNT5A* and extended into the gene body (*bumphunter*, *p* = 1.73 × 10^−4^; *DMRcate*, FDR = 2.88 × 10^−37^). The EPIC array interrogated 17 CpGs spanning the promoter region, including the TSS and two CpG islands located downstream of the TSS (Figure 1A). Significantly higher methylation was observed in primary tumours when compared to bone metastases (Figure 1B; Figure S2). *DMRcate* identified a larger region of differential methylation, including 17 CpGs, which also encompassed the region identified by *bumphunter* (11 CpGs). The region identified by *bumphunter* analysis covered position +1136 to −158, including the TSS (Figure 1B), and contained the most differentially methylated CpGs. This region was the focus of subsequent analysis, herein referred to as *WNT5A*_BH.

### 3.2 *WNT5A* is differentially methylated in cell lines of varying metastatic potential

To further investigate whether *WNT5A* is a putative epi‐driver, DNA methylation across the identified region of interest, *WNT5A*_BH, was examined in PrCa cell lines representative of a primary prostate tumour (22Rv1) and PrCa bone metastasis (PC‐3). DNA methylation of several regions encompassing *WNT5A*_BH, as shown in Figure 1A (*WNT5A*‐A, *WNT5A*‐B and *WNT5A*‐C), were examined by targeted bisulphite sequencing in both cell lines.

*WNT5A*‐A, which encompassed the TSS, had significantly higher methylation in 22Rv1 cells than PC‐3 cells, *p* < 0.01 (Figure 1C). This was reflective of what was seen in the patient paired primary tumour and metastasis samples (Figure 1B). However, DNA methylation within regions, *WNT5A*‐B and *WNT5A*‐C, which encompassed the gene body, was significantly lower in 22Rv1 cells when compared to PC‐3 cells, *p* < 0.0001 and *p* < 0.01, respectively (Figure 1D,E).

### 3.3 *WNT5A* is differentially expressed in cell lines of varying metastatic potential

**FIGURE 2:**
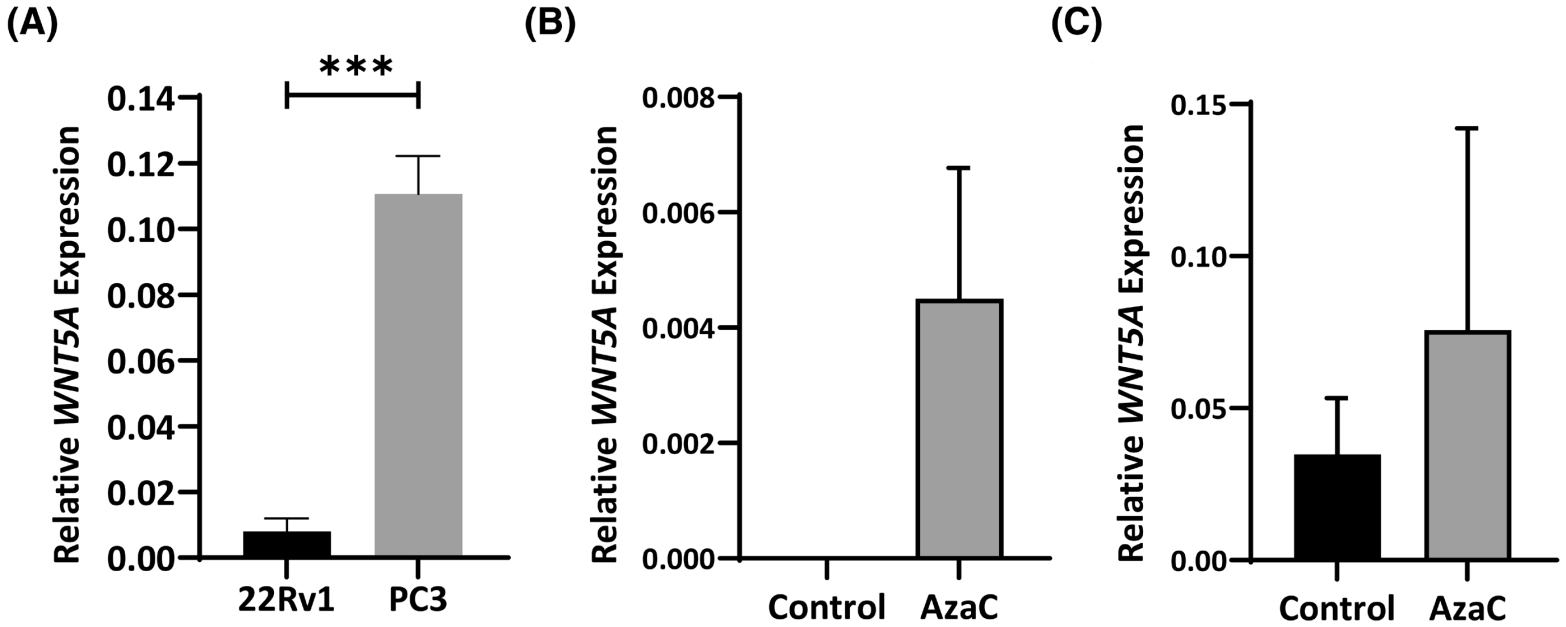
(A) *WNT5A* expression in prostate cancer cell lines. *WNT5A* mRNA levels are expressed relative to GAPDH mRNA. Values are shown as mean ± standard error (*n* = 3). (B, C) *WNT5A* expression is not altered by *5‐aza‐2′‐deoxycytidine (AzaC)* treatment. Total RNA was isolated from 22Rv1 (B) or PC‐3 (C) cells untreated or treated with AzaC. mRNA levels are expressed relative to GAPDH mRNA. Values are shown as mean ± standard error (*n* = 3). Statistical significance was determined using a Students *t*‐test, ***p < 0.001.

To determine whether differential methylation of *WNT5A* is associated with a change in gene expression, *WNT5A* expression was examined in PrCa cell lines, 22Rv1 and PC‐3. *WNT5A* expression was found to be significantly higher in the PC‐3 cells than in the 22Rv1 cells (Figure 2A). This higher expression in PC‐3 cells was associated with significantly lower DNA methylation across the TSS region (Figure 1C) and higher DNA methylation in the gene body (Figure 1D,E).

### 3.4 *WNT5A* is effectively demethylated by ***5‐aza‐2′‐deoxycytidine*** in PC‐3 and 22Rv1 cells

To further explore the relationship between DNA methylation in the *WNT5A*_BH region and *WNT5A* gene expression, mRNA levels were examined in cell lines, PC‐3 and 22Rv1, treated with demethylating agent, AzaC, to determine whether reduced DNA methylation at the *WNT5A* promoter leads to changes in *WNT5A* expression. No significant change in *WNT5A* expression was detected in either AzaC treated cell line (Figure 2B,C). However, expression trended towards increased in both cell lines, with expression being restored in AzaC treated 22Rv1 cells, which were observed to have no *WNT5A* expression in the untreated control cells (Figure 2B).

**FIGURE 3:**
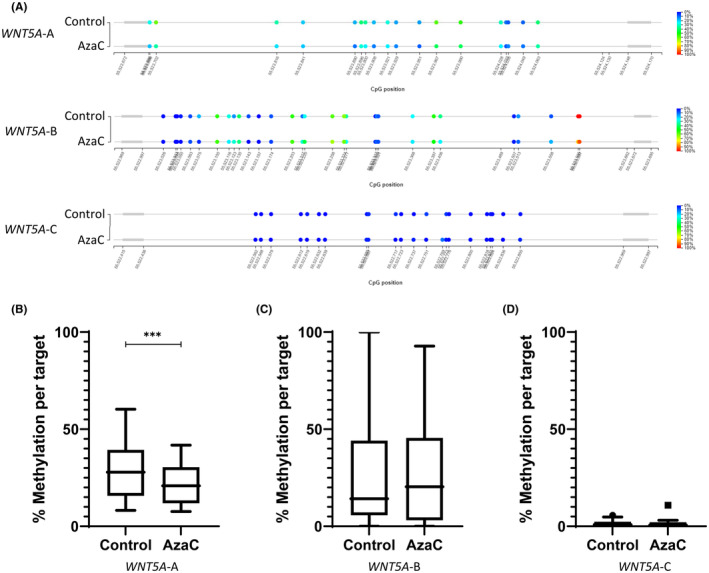
Methylation status of *WNT5A* in 22Rv1 cells. (A) Diagrammatic representation of the methylation at each CpG site within the region encompassed by *WNT5A*‐A, B and C in 22Rv1 cells. Percentage methylation is specified by colour, blue indicating a CpG is unmethylated, red indicating a CpG is methylated. (B–D) Average methylation across region encompassed by primer sets *WNT5A*‐A (B), *WNT5A*‐B (C), *WNT5A*‐C (D). Statistical significance was determined using a paired *t*‐test, ***p < 0.001.

**FIGURE 4:**
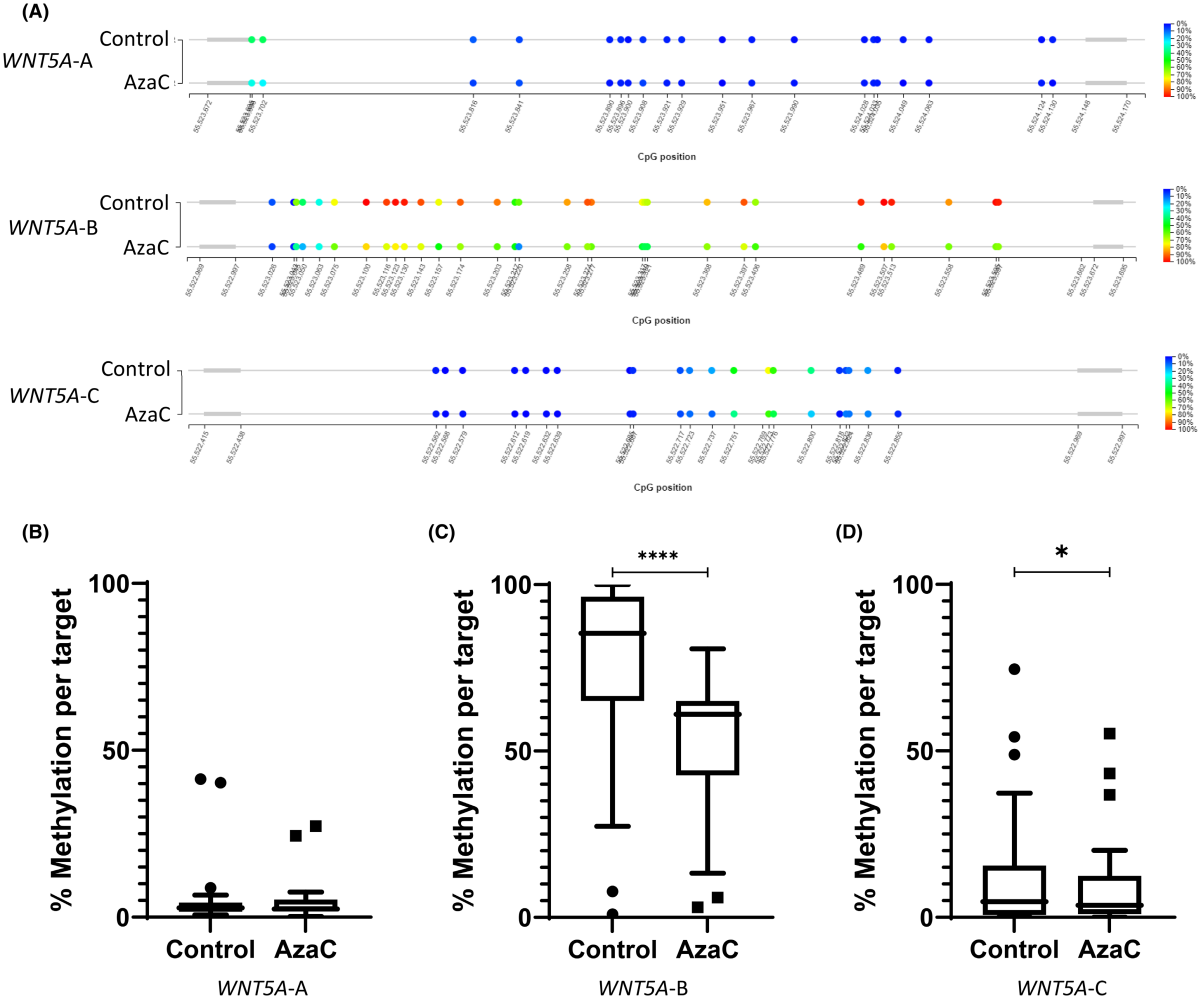
Methylation status of *WNT5A* in PC‐3 cells. (A) Diagrammatic representation of the methylation at each CpG site within the region encompassed by *WNT5A*‐A, B and C in PC‐3 cells. Percentage methylation is specified by colour, blue indicating a CpG is unmethylated, red indicating a CpG is methylated. (B–D) Average methylation across region encompassed by primer sets *WNT5A*‐A (B), *WNT5A*‐B (C), *WNT5A*‐C (D). Statistical significance was determined using a paired *t*‐test, *p < 0.05, ****p < 0.0001.

To confirm that AzaC had effectively demethylated the *WNT5A*_BH region, DNA methylation levels were investigated in the AzaC treated 22Rv1 and PC‐3 cell lines in comparison to a vehicle control (Figures 3A and 4A show representative bubble maps, respectively). The region encompassing the TSS, *WNT5A*‐A, was shown to be significantly demethylated in 22Rv1 cells, *p* < 0.001, following AzaC treatment (Figure 3B). Additionally, a significant decrease in DNA methylation was shown across the two regions situated in the gene body, *WNT5A*‐B and *WNT5A*‐C, in PC‐3 cells treated with AzaC, *p* < 0.0001 and <0.05, respectively (Figure 4C,D). Regions where DNA methylation was not significantly reduced with AzaC, had low levels of DNA methylation prior to treatment (Figure 3C,D and Figure 4B). Taken together, these results confirm that AzaC was effective in demethylating the *WNT5A*_BH region in PrCa cell lines.

Together, these data confirm that in cancer cells, differential DNA methylation of *WNT5A* is associated with changes in *WNT5A* gene expression. Particularly, high DNA methylation across the *WNT5A* TSS is associated with low *WNT5A* expression and low DNA methylation, which is paired with high DNA methylation in the gene body and higher *WNT5A* expression. Decreased methylation observed post‐AzaC treatment was also associated with restored *WNT5A* expression in 22Rv1 cells, further indicating that DNA methylation may have a functional role in regulating *WNT5A* gene expression during PrCa progression to metastatic disease.

### 3.5 *WNT5A* expression is significantly increased in bone metastases compared to other common sites of metastasis

**FIGURE 5:**
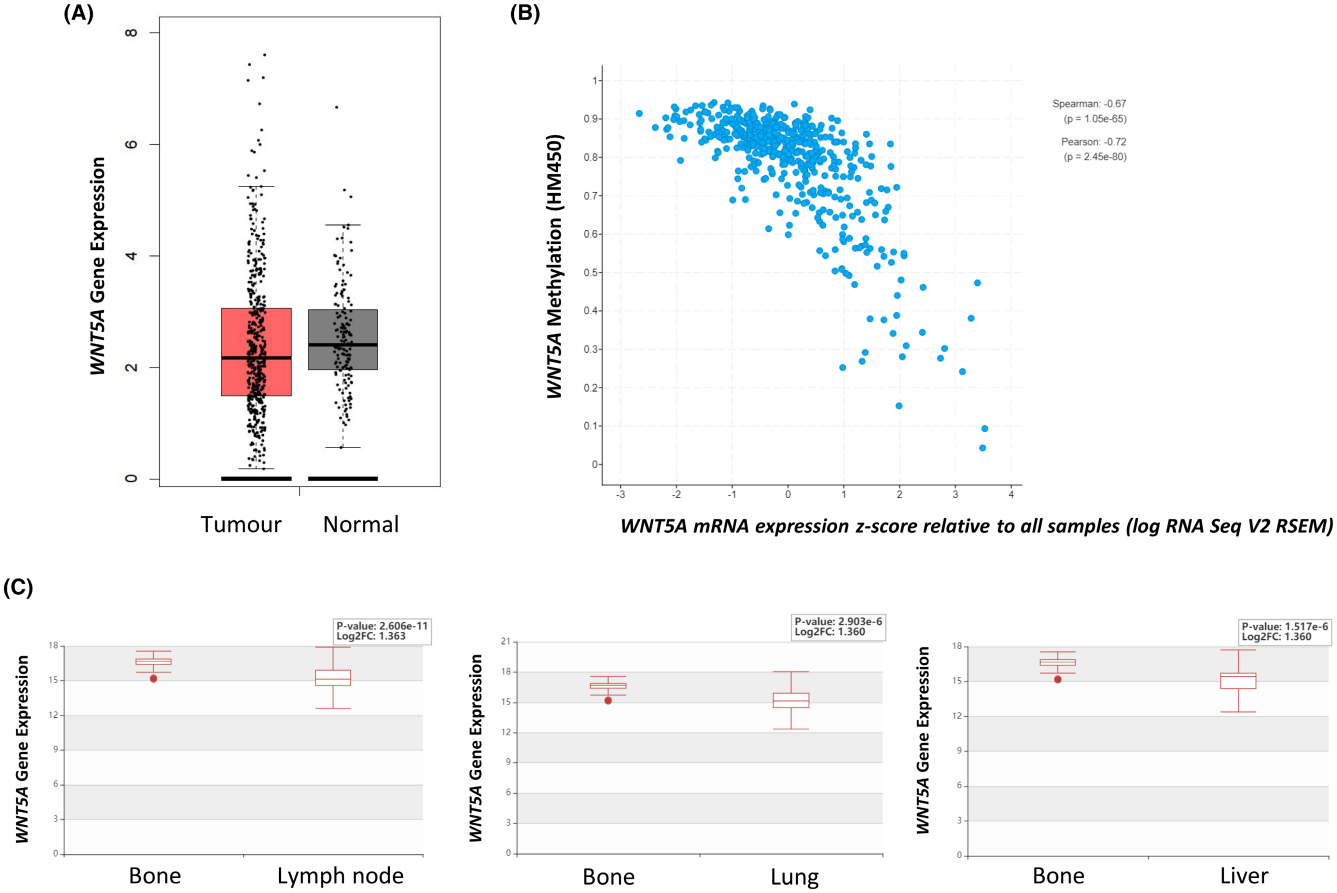
*WNT5A* expression in publicly available datasets. (A) *WNT5A* expression in tumour (*n* = 492; red) *versus* normal prostate samples (*n* = 152; grey) from TCGA‐PRAD. (B) Correlation between *WNT5A* methylation and mRNA expression in primary prostate TCGA‐PRAD tumours, taken from the cBioPortal.^20^ *WNT5A* methylation (*β*‐value) is plotted on the y‐axis, a higher value indicates higher methylation. *WNT5A* mRNA expression z‐score is plotted on the x‐axis, a higher value indicates higher expression. (C) *WNT5A* gene expression in unpaired patient samples of bone (*n* = 20), lymph node (*n* = 69), lung (*n* = 22) and liver (*n* = 21) metastases. Data and images were taken from the Human Cancer Metastasis Database in 2021 (EXP00337‐9).^21^

*WNT5A* methylation and expression was interrogated in the TCGA‐PRAD tumours (*n* = 492). Analysis found similar *WNT5A* expression in tumour *versus* normal prostate samples (*n* = 152; Figure 5A).^19^ However, higher *WNT5A* expression was found to be associated with shorter disease‐free survival (Figure S3).^19^ Furthermore, comparison of methylation levels to gene expression of *WNT5A*, indicated a strong negative correlation between the two (Figure 5B; taken from the cBioPortal^20^). Specifically, decreased methylation was correlated with an increase in expression which indicates that in our paired patient samples, changes in methylation may be accompanied by changes in gene expression, which we observed in our cell line analyses. This was consistent with publicly available data previously available from the Human Cancer Metastasis Database.^21^ *WNT5A* expression was found to be significantly higher in bone metastases compared to other sites of metastasis, such as the lymph node, lung and liver (Figure 5C).

## 4 DISCUSSION

Here, we utilised the power of paired patient samples^22^ to identify shared key methylation changes between primary prostate tumours and bone metastases. The principle underpinning the ‘paired sample’ approach is that although potentially thousands of molecular changes are likely to be identified between the paired samples, only those shared between individuals are likely to be the key drivers of metastatic survival and growth in these bony lesions.^22^ Notably, patterns of DNA methylation clustered on an individual basis instead of by sample type (primary vs. metastatic; Figure S1), which is consistent with previous studies.^23^ Patient samples showed strong evidence for methylation differences associated with disease state. We identified a list of DMRs associated with 3436 genes found to be consistently differentially methylated in bone metastases when compared to primary prostate tumours. One of the most significant regions of differential methylation identified was an approximately 1 kb region within the *WNT5A* promoter, which was significantly demethylated in bone metastases when compared to primary prostate tumours.

Aberrant *WNT5A* DNA methylation has been reported throughout PrCa disease progression, with the *WNT5A* promoter shown to be hypermethylated in primary PrCa tumours when compared to benign prostatic hyperplasia^24^ and hypomethylated when compared to benign tissue.^25^ DNA methylation in the identified *WNT5A* region was further investigated in cell line models of primary prostate tumour and bone metastasis, 22Rv1 and PC‐3 cells, respectively. The methylation patterns across the TSS were found to resemble the methylation patterns of the primary patient samples. It was further found that differential *WNT5A* DNA methylation was negatively correlated with *WNT5A* gene expression in the cell lines. Consistent with our findings here, it has previously been reported that when hypomethylated, *WNT5A* is upregulated when compared to normal cells.^25^ In a multi‐omic approach to assessing the proteome in 949 human cancer cell lines, Goncalves and colleagues (2022) also reported increased *WNT5A* expression in cell lines of higher metastatic potential (PC‐3 > VCaP>LNCaP>22Rv1).^26^ In addition, here, AzaC‐induced demethylation was associated with a shift of *WNT5A* expression in both cell lines, however, this change did not reach significance and may indicate that AzaC is not able to sufficiently demethylate *WNT5A*. Although, another study found that treatment of a normal prostate cell line with AzaC resulted in partial loss of methylation and was shown to increase mRNA expression.^27^

*WNT5A* encodes a secreted signalling protein which plays an essential role in the regulation of developmental pathways during embryonic development.^28^ Increased *WNT5A* expression has been shown to induce epithelial to mesenchymal transition across multiple cancer types (breast, lung and colon), and in cell lines taken from hepatocellular and ovarian cancer.^29^, ^30^ Additionally, changes in gene and protein expression of WNT5A have been consistently implicated in both primary PrCa disease progression and metastasis to the bone, however, it is debated as to whether an increase in expression promotes or inhibits disease progression.^31^, ^32^, ^33^, ^34^, ^35^, ^36^ Analysis of publicly available datasets revealed that higher *WNT5A* expression is associated with shorter disease‐free survival and increased expression is observed in bone metastases compared to other metastatic sites. However, there is a dearth of publicly available databases that report analysis of paired primary and metastatic samples, and so no comparative data were identified to provide a true comparison. Knockdown of *WNT5A* and other downstream Wnt signalling receptors in in vitro models, which mimic the bone microenvironment, significantly reduces PrCa cell line invasiveness in PC‐3 and 22Rv1 cells.^35^, ^37^ Additionally, upregulation of *WNT5A* has been shown to enhance cell motility, migration and invasion,^25^, ^38^ and in vitro, increases cell migration potential towards bone, indicating its potential role as a chemoattractant.^37^ Conversely, contrary to our findings, a study also employing PC‐3 cells found that *WNT5A* induced dormancy of PrCa cells in vitro, in a reversible manner, and inhibited bone metastasis in vivo.^34^ Likewise, a study by Thiele and colleagues (2015) also found that *WNT5A* overexpression decreased cell proliferation and migration, and increased cancer cell apoptosis.^31^ Taken together, these data reveal that the role of *WNT5A* expression in PrCa carcinogenesis is complex.

It has been established in several previous studies that methylation of *WNT5A* is associated with gene expression, and that *WNT5A* is likely to be epigenetically regulated through methylation changes at or around the promoter region. In PrCa, converse to what we have shown here, hypomethylation of *WNT5A* has been observed in primary prostate tumours^27^ most notably, in a study of 100 independent metastatic castration resistant PrCa tumours by Zhao et al., (2020), *WNT5A* was hypomethylated in these tumours and methylation was negatively correlated with *WNT5A* expression (*p* = 7.63 × 10^−6^),^39^ which supports our findings. Given its role as a tumour suppressor gene in multiple cancer types, methylation at the *WNT5A* promoter can also be paired with decreased expression,^40^, ^41^, ^42^ and increased DNA methylation is associated with multiple haematological malignancies,^40^, ^41^ oesophageal squamous cell carcinoma^42^ and ovarian cancer.^43^ Further, in colorectal cancer, *WNT5A* methylation is associated with tumour microsatellite instability, and therefore is particularly prone to replication errors and is predictive of response to chemotherapy and immunotherapy.^44^ In many cases, cell lines of these malignancies show decreased methylation and increased *WNT5A* expression when treated with the demethylating agent, AzaC.^27^, ^40^, ^42^, ^43^, ^45^

Previous studies have demonstrated that small sample sizes of paired patient samples are sufficiently powered to identify differential methylation across groups, compared to when non‐paired samples are used.^23^, ^46^ Studies using a paired sample design, which was employed here, provide the power to identify candidate genes,^22^ however further validation in a larger sample size and/or an in vitro model are still required. These paired samples are a rare resource, however, their utility is restricted by sample availability. In Australia, biopsies aren't commonly used to diagnose bone metastasis and, when they are, it is often years after the initial prostate biopsy. The data presented here indicates that the decreased pattern of methylation observed in bone metastasis may be associated with an increase in *WNT5A* expression. However, this hypothesis was not tested in the patient samples. This is because it is difficult to extract quality RNA from FFPE tissue samples derived from the bone. Nucleic acids are often degraded during the fixation and embedding process and a calcified bone sample adds extra complexity.^47^, ^48^ Thus, we were unable to generate accurate representation of RNA expression changes associated with any identified DNA methylation changes. Utilising cell line models, as we have here, is an effective alternative to investigating expression changes that may be associated with DNA methylation changes.

The majority of PrCa mortality burden lies with metastasis, particularly bone metastasis. Additionally, there is significant disease burden caused by overdiagnosis and overtreatment of PrCa. Therefore, identifying epi‐drivers that could aid in the development of a putative diagnostic test for the risk of metastasis, would allow for more informed clinical decision making. Consequently, identifying potential drivers of PrCa progression to bone metastasis is imperative to providing novel therapeutic targets. Taken together with the literature, the data presented here suggest that the DNA methylation changes in *WNT5A* are associated with *WNT5A* expression, which shows great promise as a potential epi‐driver of metastasis. Our study demonstrates the utility of this combined patient paired sample and cell line model approach in identifying potential epi‐drivers of metastasis. More broadly, aberrations in WNT signalling pathway genes have historically been considered for cancer treatments but remain unavailable due to side effects.^49^ WNT5A inhibitors have been shown to effectively suppress melanoma cell invasion in vitro by blocking WNT5A signalling,^50^, ^51^ and have been identified as potential therapeutic options for ovarian cancer,^52^ however, validation of their clinical utility in vivo is urgently required. Current WNT5A inhibitors are non‐specific to WNT5A which may mean that ‘normal’ WNT signalling may also be compromised. Overall, further investigation into the pathways through which *WNT5A* dysregulation may induce metastasis is an obvious next step.

## AUTHOR CONTRIBUTIONS

**Emma J. Wilkinson:** Data curation (lead); formal analysis (lead); investigation (lead); validation (lead); visualization (equal); writing – original draft (equal); writing – review and editing (equal). **Kelsie Raspin:** Data curation (supporting); formal analysis (supporting); investigation (supporting); methodology (supporting); project administration (equal); resources (supporting); supervision (supporting); visualization (equal); writing – original draft (equal); writing – review and editing (equal). **Roslyn C. Malley:** Data curation (supporting); resources (supporting); writing – review and editing (supporting). **Shaun Donovan:** Data curation (supporting); resources (supporting); writing – review and editing (supporting). **Louise M. Nott:** Conceptualization (supporting); resources (supporting); writing – review and editing (supporting). **Adele F. Holloway:** Conceptualization (equal); funding acquisition (equal); methodology (equal); resources (equal); software (equal); supervision (equal); writing – review and editing (equal). **Joanne L. Dickinson:** Conceptualization (equal); funding acquisition (equal); methodology (equal); project administration (lead); resources (equal); software (equal); supervision (equal); writing – review and editing (equal).

## FUNDING INFORMATION

Andree Greenwood Secondary Breast Cancer Fund; the Hobart Police Charity Trust; the Royal Hobart Hospital Research Foundation; the Cancer Council Tasmania; Cuthbertson Brothers Elite Postgraduate Scholarship; Cancer Council Tasmania Joy and Robert Coghlan/College of Health and Medicine Postdoctoral Research Fellowship; Select Foundation Cancer Research Fellowship and an Australian Research Council Future Fellowship.

## CONFLICT OF INTEREST STATEMENT

The authors declare no conflict of interest.

## ETHICS STATEMENT

This study was ethically approved by the University of Tasmania's Human Research Ethics Committee (H0020219), with a waiver of consent granted to obtain patient tissue samples.

## Supporting information


Data S1.

## Data Availability

EPIC array data generated in this study are available in BioStudies under accession number S‐BSST1520. Additional information can be found in the supplementary material.

## References

[1] Howlader N , Noone AM , Krapcho M , et al. (eds) SEER cancer statistics review, 1975‐2017. National Cancer Institute; 2020. https://seer.cancer.gov/csr/1975_2017/

[2] Hernandez RK , Wade SW , Reich A , Pirolli M , Liede A , Lyman GH . Incidence of bone metastases in patients with solid tumors: analysis of oncology electronic medical records in the United States. BMC Cancer. 2018;18(1):44. 29306325 10.1186/s12885-017-3922-0 PMC5756362

[3] Gandaglia G , Abdollah F , Schiffmann J , et al. Distribution of metastatic sites in patients with prostate cancer: a population‐based analysis. Prostate. 2014;74(2):210‐216. 24132735 10.1002/pros.22742

[4] Nørgaard M , Jensen A , Jacobsen JB , Cetin K , Fryzek JP , Sørensen HT . Skeletal related events, bone metastasis and survival of prostate cancer: a population based cohort study in Denmark (1999 to 2007). J Urol. 2010;184(1):162‐167. 20483155 10.1016/j.juro.2010.03.034

[5] Wang L , Lu B , He M , Wang Y , Wang Z , Du L . Prostate cancer incidence and mortality: global status and temporal trends in 89 countries from 2000 to 2019. Front Public Health. 2022;10:811044. 35252092 10.3389/fpubh.2022.811044 PMC8888523

[6] Schmid‐Alliana A , Schmid‐Antomarchi H , Al‐Sahlanee R , Lagadec P , Scimeca JC , Verron E . Understanding the progression of bone metastases to identify novel therapeutic targets. Int J Mol Sci. 2018;19(1):148. 29300334 10.3390/ijms19010148 PMC5796097

[7] Chatterjee A , Rodger EJ , Eccles MR . Epigenetic drivers of tumourigenesis and cancer metastasis. Semin Cancer Biol. 2018;51:149‐159. 28807546 10.1016/j.semcancer.2017.08.004

[8] Rycaj K , Tang DG . Molecular determinants of prostate cancer metastasis. Oncotarget. 2017;8(50):88211‐88231. 29152153 10.18632/oncotarget.21085 PMC5675705

[9] Patel R , Brzezinska EA , Repiscak P , et al. Activation of β‐catenin cooperates with loss of Pten to drive AR‐independent castration‐resistant prostate cancer. Cancer Res. 2020;80(3):576‐590. 31719098 10.1158/0008-5472.CAN-19-1684

[10] Ayres Pereira M , Chio IIC . Metastasis in pancreatic ductal adenocarcinoma: current standing and methodologies. Genes (Basel). 2019;11(1):6. 31861620 10.3390/genes11010006 PMC7016631

[11] Mundbjerg K , Chopra S , Alemozaffar M , et al. Identifying aggressive prostate cancer foci using a DNA methylation classifier. Genome Biol. 2017;18(1):3. 28081708 10.1186/s13059-016-1129-3 PMC5234101

[12] Wilkinson EJ , Woodworth AM , Parker M , et al. Epigenetic regulation of the ITGB4 gene in prostate cancer. Exp Cell Res. 2020;392(2):112055. 32376286 10.1016/j.yexcr.2020.112055

[13] Jaffe AE , Murakami P , Lee H , et al. Bump hunting to identify differentially methylated regions in epigenetic epidemiology studies. Int J Epidemiol. 2012;41(1):200‐209. 22422453 10.1093/ije/dyr238 PMC3304533

[14] Peters TJ , Buckley MJ , Statham AL , Pidsley R , Samaras K , et al. De novo identification of differentially methylated regions in the human genome. Epigenetics Chromatin. 2015;8(1):6. 25972926 10.1186/1756-8935-8-6 PMC4429355

[15] Cavalcante RG , Sartor MA . annotatr: genomic regions in context. Bioinformatics. 2017;33(15):2381‐2383. 28369316 10.1093/bioinformatics/btx183 PMC5860117

[16] Wickham H , François R , Henry L , Müller K . dplyr: A Grammar of Data Manipulation . 2020. R package version 0.8.5. https://CRAN.R‐project.org/package=dplyr

[17] Krainer J , Weinhäusel A , Hanak K , et al. EPIC‐TABSAT: Analysis tool for targeted bisulfite sequencing experiments and array‐based methylation studies. Nucleic Acids Res. 2019;47(W1):W166‐W170. 31106358 10.1093/nar/gkz398 PMC6602470

[18] Asem MS , Buechler S , Wates RB , Miller DL , Stack MS . Wnt5a signaling in cancer. Cancers. 2016;8(9):79. 27571105 10.3390/cancers8090079 PMC5040981

[19] Tang Z , Li C , Kang B , Gao G , Li C , Zhang Z . GEPIA: a web server for cancer and normal gene expression profiling and interactive analyses. Nucleic Acids Res. 2017;45(W1):98‐102. 10.1093/nar/gkx247 PMC5570223 28407145

[20] Gao J , Aksoy BA , Dogrusoz U , et al. Integrative analysis of complex cancer genomics and clinical profiles using the cBioPortal. Sci Signal. 2013;6(269):pl1. 23550210 10.1126/scisignal.2004088 PMC4160307

[21] Zheng G , Ma Y , Zou Y , Yin A , Li W , Dong D . HCMDB: the human cancer metastasis database. Nucleic Acids Res. 2018;46(D1):950‐955. 10.1093/nar/gkx1008 PMC5753185 29088455

[22] Stevens JR , Herrick JS , Wolff RK , Slattery ML . Power in pairs: assessing the statistical value of paired samples in tests for differential expression. BMC Genomics. 2018;19(1):953. 30572829 10.1186/s12864-018-5236-2 PMC6302489

[23] Aryee MJ , Liu W , Engelmann JC , et al. DNA methylation alterations exhibit intraindividual stability and interindividual heterogeneity in prostate cancer metastases. Sci Transl Med. 2013;5(169):169ra10. 10.1126/scitranslmed.3005211 PMC3577373 23345608

[24] Vasiljević N , Ahmad AS , Carter PD , et al. DNA methylation of PITX2 predicts poor survival in men with prostate cancer. Biomark Med. 2014;8(9):1143‐1150. 25402584 10.2217/bmm.14.41

[25] Wang Q , Symes AJ , Kane CA , et al. A novel role for Wnt/Ca2+ signaling in actin cytoskeleton remodeling and cell motility in prostate cancer. PLoS One. 2010;5(5):e10456. 20454608 10.1371/journal.pone.0010456 PMC2864254

[26] Gonçalves E , Poulos RC , Cai Z , et al. Pan‐cancer proteomic map of 949 human cell lines. Cancer Cell. 2022;40(8):835‐849. 35839778 10.1016/j.ccell.2022.06.010 PMC9387775

[27] Wang Q , Williamson M , Bott S , et al. Hypomethylation of WNT5A, CRIP1 and S100P in prostate cancer. Oncogene. 2007;26(45):6560‐6565. 17486081 10.1038/sj.onc.1210472

[28] Kumawat K , Gosens R . WNT‐5A: signaling and functions in health and disease. Cell Mol Life Sci. 2016;73(3):567‐587. 26514730 10.1007/s00018-015-2076-y PMC4713724

[29] Gujral TS , Chan M , Peshkin L , Sorger PK , Kirschner MW , MacBeath G . A noncanonical Frizzled2 pathway regulates epithelial‐mesenchymal transition and metastasis. Cell. 2014;159(4):844‐856. 25417160 10.1016/j.cell.2014.10.032 PMC4243058

[30] Henry C , Llamosas E , Knipprath‐Meszaros A , et al. Targeting the ROR1 and ROR2 receptors in epithelial ovarian cancer inhibits cell migration and invasion. Oncotarget. 2015;6(37):40310‐40326. 26515598 10.18632/oncotarget.5643 PMC4741897

[31] Thiele S , Göbel A , Rachner TD , et al. WNT5A has anti‐prostate cancer effects in vitro and reduces tumor growth in the skeleton in vivo. J Bone Miner Res. 2015;30(3):471‐480. 25224731 10.1002/jbmr.2362

[32] Carneiro I , Quintela‐Vieira F , Lobo J , et al. Expression of EMT‐related genes CAMK2N1 and WNT5A is increased in locally invasive and metastatic prostate cancer. J Cancer. 2019;10(24):5915‐5925. 31762801 10.7150/jca.34564 PMC6856586

[33] Syed Khaja AS , Helczynski L , Edsjö A , et al. Elevated level of Wnt5a protein in localized prostate cancer tissue is associated with better outcome. PLoS One. 2011;6(10):e26539. 22039506 10.1371/journal.pone.0026539 PMC3200334

[34] Ren D , Dai Y , Yang Q , et al. Wnt5a induces and maintains prostate cancer cells dormancy in bone. J Exp Med. 2019;216(2):428‐449. 30593464 10.1084/jem.20180661 PMC6363426

[35] Wang Y , Singhal U , Qiao Y , et al. Wnt signaling drives prostate cancer bone metastatic tropism and invasion. Transl Oncol. 2020;13(4):100747. 32217460 10.1016/j.tranon.2020.100747 PMC7109463

[36] Dai J , Hall CL , Escara‐Wilke J , Mizokami A , Keller JM , Keller ET . Prostate cancer induces bone metastasis through Wnt‐induced bone morphogenetic protein‐dependent and independent mechanisms. Cancer Res. 2008;68(14):5785‐5794. 18632632 10.1158/0008-5472.CAN-07-6541 PMC4432935

[37] Jin F , Qu X , Fan Q , et al. Regulation of prostate cancer cell migration toward bone marrow stromal cell‐conditioned medium by Wnt5a signaling. Mol Med Rep. 2013;8(5):1486‐1492. 24064566 10.3892/mmr.2013.1698

[38] Yamamoto H , Oue N , Sato A , et al. Wnt5a signaling is involved in the aggressiveness of prostate cancer and expression of metalloproteinase. Oncogene. 2010;29(14):2036‐2046. 20101234 10.1038/onc.2009.496

[39] Zhao SG , Chen WS , Li H , et al. The DNA methylation landscape of advanced prostate cancer. Nat Genet. 2020;52(8):778‐789. 32661416 10.1038/s41588-020-0648-8 PMC7454228

[40] Roman‐Gomez J , Jimenez‐Velasco A , Cordeu L , et al. WNT5A, a putative tumour suppressor of lymphoid malignancies, is inactivated by aberrant methylation in acute lymphoblastic leukaemia. Eur J Cancer. 2007;43(18):2736‐2746. 18032022 10.1016/j.ejca.2007.10.004

[41] Ying J , Li H , Chen YW , Srivastava G , Gao Z , Tao Q . WNT5A is epigenetically silenced in hematologic malignancies and inhibits leukemia cell growth as a tumor suppressor. Blood. 2007;110(12):4130‐4132. 18024799 10.1182/blood-2007-06-094870

[42] Li J , Ying J , Fan Y , et al. WNT5A antagonizes WNT/β‐catenin signaling and is frequently silenced by promoter CpG methylation in esophageal squamous cell carcinoma. Cancer Biol Ther. 2010;10(6):617‐624. 20603606 10.4161/cbt.10.6.12609

[43] Jin P , Song Y , Yu G . The role of abnormal methylation of Wnt5a gene promoter regions in human epithelial ovarian cancer: a clinical and experimental study. Anal Cell Pathol. 2018;2018:6567081. 10.1155/2018/6567081 PMC6069701 30079293

[44] Rawson J , Mrkonjic M , Daftary D , et al. Promoter methylation of Wnt5a is associated with microsatellite instability and BRAF V600E mutation in two large populations of colorectal cancer patients. Br J Cancer. 2011;104(12):1906‐1912. 21587258 10.1038/bjc.2011.165 PMC3111198

[45] Ying J , Li H , Yu J , et al. WNT5A exhibits tumor‐suppressive activity through antagonizing the WNT/β‐catenin signaling, and is frequently methylated in colorectal cancer. Clin Cancer Res. 2008;14(1):55‐61. 18172252 10.1158/1078-0432.CCR-07-1644

[46] Chatterjee A , Stockwell PA , Ahn A , Rodger EJ , Leichter AL , Eccles MR . Genome‐wide methylation sequencing of paired primary and metastatic cell lines identifies common DNA methylation changes and a role for EBF3 as a candidate epigenetic driver of melanoma metastasis. Oncotarget. 2017;8(4):6085‐6101. 28030832 10.18632/oncotarget.14042 PMC5351615

[47] Feldman MY . Reactions of nucleic acids and NucleoDroteins with Formaldehyde. In: Pumpiansky L , Davidson JN , Cohn WE , eds. Progress in Nucleic Acid Research and Molecular Biology. Academic Press; 1973:1‐49. 10.1016/s0079-6603(08)60099-9 4573489

[48] Brutlag D , Schlehuber C , Bonner J . Properties of formaldehyde‐treated nucleohistone. Biochemistry. 1969;8(8):3214‐3218. 5809221 10.1021/bi00836a013

[49] Park WJ , Kim MJ . A new wave of targeting 'Undruggable' Wnt signaling for cancer therapy: challenges and opportunities. Cells. 2023;12(8):1110. 37190019 10.3390/cells12081110 PMC10136927

[50] Radaszkiewicz T , Nosková M , Gömöryová K , et al. RNF43 inhibits WNT5A‐driven signaling and suppresses melanoma invasion and resistance to the targeted therapy. elife. 2021;10:e65759. 34702444 10.7554/eLife.65759 PMC8550759

[51] Jenei V , Sherwood V , Howlin J , et al. A t‐butyloxycarbonyl‐modified Wnt5a‐derived hexapeptide functions as a potent antagonist of Wnt5a‐dependent melanoma cell invasion. Proc Natl Acad Sci USA. 2009;106(46):19473‐19478. 19901340 10.1073/pnas.0909409106 PMC2780806

[52] Zhou W , Mei J , Gu D , et al. Wnt5a: a promising therapeutic target in ovarian cancer. Pathol Res Pract. 2021;219:153348. 33540373 10.1016/j.prp.2021.153348

