# Supplementary material for: WNT5A is a putative epi‐driver of prostate cancer metastasis to the bone
Source: Cancer Med. 2024 Aug 20;13(16):e70122. doi: 10.1002/cam4.70122 (PMC11335815; doi:10.1002/cam4.70122)
Supplement: Supplementary file 1 — Data S1. [file CAM4-13-e70122-s001.docx]

***WNT5A* is a putative epi-driver of prostate cancer metastasis to the bone**

Emma J Wilkinson^1,2, #^, Kelsie Raspin^2, #^, Roslyn C Malley^1,3,4^, Shaun Donovan^4^, Louise M Nott^2,3,5^, Adele F Holloway^1, ^^, Joanne L Dickinson^2, ^,*^

# ^1^ Tasmanian School of Medicine, University of Tasmania, Hobart, Tasmania, Australia

^2^ Menzies Institute for Medical Research, University of Tasmania, Hobart, Tasmania, Australia

^3^ Royal Hobart Hospital, Hobart, Tasmania, Australia

^4^ Sonic Healthcare, Hobart, Tasmania, Australia

^5^ Icon Cancer Centre, Hobart, Tasmania, Australia

^#^ Joint first authors.

^ Joint senior authors.

* To whom correspondence should be addressed. Tel: +61 3 6226 7622; Email: [jo.dickinson@utas.ed.au](mailto:jo.dickinson@utas.ed.au); ORCHID: 0000-0003-4621-1703.

**SUPPLEMENTARY FIGURES**


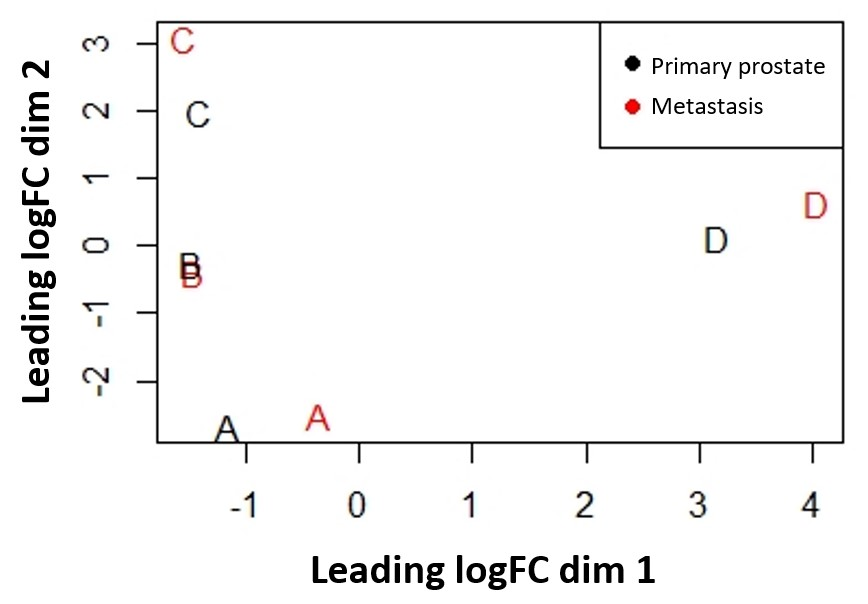


**Supplementary Figure 1.** Multidimensional scaling plot of EPIC array M-values of paired primary PrCa and metastatic bone samples.

**Supplementary Figure 2.** Heatmap of the methylation status of the 17 CpG sites within the significantly differentially methylated region of *WNT5A* identified using DMRcate. The heatmap was made using Morpheus, <https://software.broadinstitute.org/morpheus>.

**
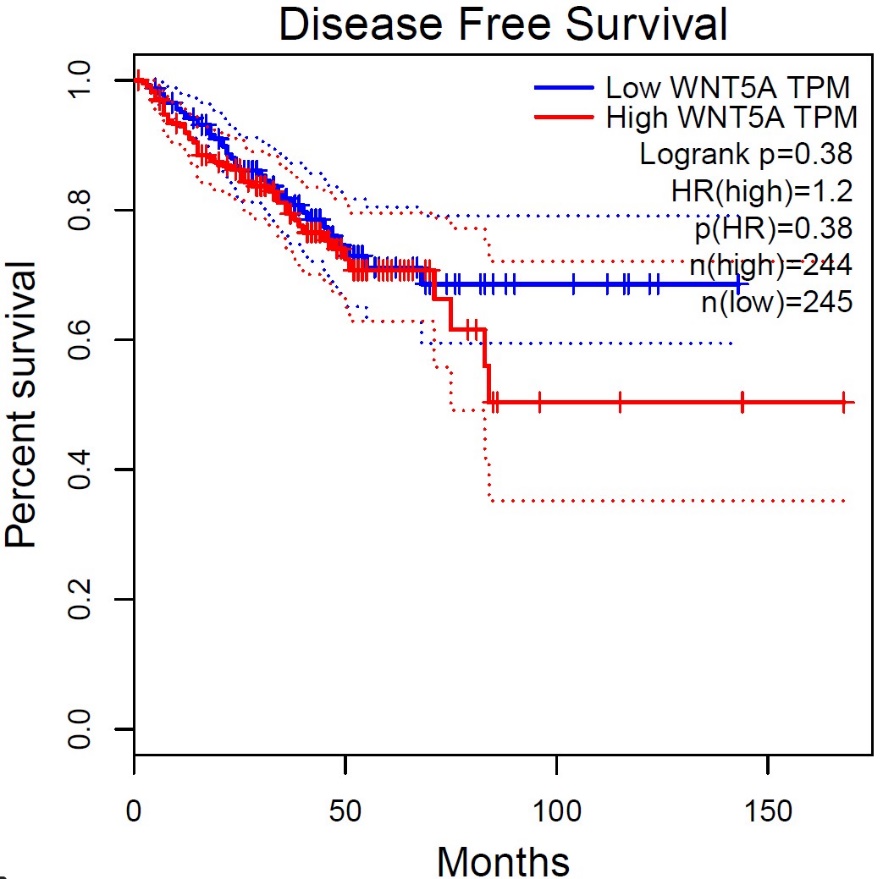
**

**Supplementary Figure 3.** Disease free survival based on *WNT5A* expression. TCGA-PRAD tumours with high expression (n=245) had shorter disease-free survival compared with prostate tumours with low *WNT5A* expression (n=244) [1].

**SUPPLEMENTARY TABLES**

**Supplementary Table 1.** Top ten most significantly differentially methylated regions identified using bumphunter.

| **Chromosome** | **Start** | **End** | **Number of CpGs** | **p value** | **Gene** |
| --- | --- | --- | --- | --- | --- |
| 11 | 2397201 | 2397831 | 17 | 4.27x10^-5^ | *CD81* |
| 21 | 34775001 | 34775045 | 5 | 7.31x10^-5^ | *IFNGR2* |
| 17 | 74641167 | 74641294 | 4 | 8.40x10^-5^ | *ST6FALNAC1* |
| 4 | 1202653 | 1203653 | 16 | 9.07x10^-5^ | *CTBP1* |
| 19 | 51359602 | 51359917 | 3 | 0.00102 | *KLK3* |
| 21 | 38076709 | 38079615 | 15 | 0.000111 | *SIM2* |
| 17 | 76991208 | 76991378 | 4 | 0.000128 | *CANT1* |
| 12 | 52473422 | 52473731 | 4 | 0.00013 | *OR7E47P* |
| 12 | 115134201 | 115135003 | 15 | 0.000133 | *N/A* |
| 3 | 55522835 | 55524129 | 11 | 0.000173 | *WNT5A* |
| N/A indicates the region with no gene annotation. | | | | | |

**Supplementary Table 2.** Top ten most significantly differentially methylated regions identified using DMRcate.

| **Chromosome** | **Start** | **End** | **Number of CpGs** | **p value** | **Gene** |
| --- | --- | --- | --- | --- | --- |
| 15 | 101418967 | 101420185 | 16 | 3.68x10^-59^ | *ALDH1A3* |
| 12 | 115131223 | 115136308 | 61 | 5.33x10^-46^ | *N/A* |
| 11 | 2397201 | 2398533 | 31 | 4.03x10^-44^ | *CD81* |
| 6 | 137365300 | 137367237 | 17 | 2.16x10^-43^ | *IL20RA* |
| 17 | 74639731 | 74640078 | 6 | 9.67x10^-40^ | *ST6GALNAC1* |
| 3 | 55522301 | 55525049 | 17 | 2.88x10^-37^ | *WNT5A* |
| 20 | 56265517 | 56268070 | 14 | 1.95x10^-34^ | *PMEPA1* |
| 18 | 56528679 | 56530789 | 15 | 1.36x10^-33^ | *ZNF532* |
| 1 | 3276354 | 3277623 | 12 | 4.75x10^-33^ | *N/A* |
| 17 | 27944453 | 27945882 | 8 | 3.17x10^-32^ | *CORO6* |
| N/A indicates the region with no gene annotation. | | | | | |

**REFERENCES**

1. Tang, Z., *et al.*, GEPIA: a web server for cancer and normal gene expression profiling and interactive analyses. *Nucleic Acids Res*, 2017. 45(1): p. 98-102.
